# Production of Polygalacturonases by *Aspergillus* Section *Nigri *Strains in a Fixed Bed Reactor

**DOI:** 10.3390/molecules18021660

**Published:** 2013-01-28

**Authors:** Marília Maciel, Cristiane Ottoni, Cledir Santos, Nelson Lima, Keila Moreira, Cristina Souza-Motta

**Affiliations:** 1 Mycology Department, Federal University of Pernambuco, Cidade Universitária, Recife 50670-420, Pernambuco, Brazil; E-Mail: lilomaciel@hotmail.com; 2 IBB-Institute for Biotechnology and Bioengineering, Centre of Biological Engineering, Micoteca da Universidade do Minho, University of Minho, Campus of Gualtar, Braga 4710-057, Portugal; E-Mails: cristiane.ottoni@deb.uminho.pt (C.O.); cledir.santos@deb.uminho.pt (C.S.); nelson@ie.uminho.pt (N.L.); 3 Academic Unit of Garanhuns, Federal Rural University of Pernambuco, Garanhuns 55292-270, Pernambuco, Brazil; E-Mail: moreiralab@yahoo.com.br

**Keywords:** *Aspergillusniger*, orange peel, immobilization cells, polygalacturonases, fixed bed reactor

## Abstract

Polygalacturonases (PG) are pectinolytic enzymes that have technological, functional and biological applications in food processing, fruit ripening and plant-fungus interactions, respectively. In the present, a microtitre plate methodology was used for rapid screening of 61 isolates of fungi from *Aspergillus* section *Nigri* to assess production of endo- and exo-PG. Studies of scale-up were carried out in a fixed bed reactor operated under different parameters using the best producer strain immobilised in orange peels. Four experiments were conducted under the following conditions: the immobilised cells without aeration; immobilised cells with aeration; immobilised cells with aeration and added pectin; and free cells with aeration. The fermentation was performed for 168 h with removal of sample every 24 h. *Aspergillus niger* strain URM 5162 showed the highest PG production. The results obtained indicated that the maximum endo- and exo-PG activities (1.18 U·mL^−1^ and 4.11 U·mL^−1^, respectively) were obtained when the reactor was operating without aeration. The microtitre plate method is a simple way to screen fungal isolates for PG activity detection. The fixed bed reactor with orange peel support and using *A*. *niger* URM 5162 is a promising process for PG production at the industrial level.

## 1. Introduction

Orange juice is an important economic contributor to the Brazilian Gross Domestic Product (GDP). Approximately 85% of the total amount of orange juice produced in Brazil is for exportation, which makes Brazil responsible for more than 50% of the world production. Brazilian orange juice production is greater than that of coffee, beef, chicken and sugar [1]. However, the process generates large amounts of residues composed especially of peel and segment membranes. These are rich in soluble and insoluble carbohydrates [2]: peel contains approximately 17% soluble sugar, 9% cellulose, 10% hemicellulose and 42% pectin as the main components [3].

Orange peels as a carbon source and inducer of pectinase production are very attractive to the enzyme industry [2]. Conversion of pectin to soluble sugars is possible through enzymatic reactions catalysed by pectinolytic enzymes which are common in fungi, such as the pectin lyase (PL) (EC 4.2.1.10), pectin methylesterase (PME) (EC 3.2.1.11) and polygalacturonase (PG) (EC 3.2.1.15) [4]. PG have hydrolysis as the main function and are the most used in industrial processes. Indeed, fungi are employed to produce PG in a large scale under acidic conditions. Moreover, fungal PG are most useful because of their high activity and optimal activity at low pH which is suitable for most potential fruit and vegetable substrates [5].

Approximately 25% of the enzymes sold in the global market of the food industry are pectinases [6,7] where the predominant industrial application is juice and wine clarification. Clarification is achieved by promoting pectin hydrolysis and polysaccharide solubilisation which promotes decreasing viscosity and the agglomeration of suspend solid particles, which can then be removed by filtration or centrifugation [8,9].

Pectinases can often be produced at high concentrations by strains of filamentous fungi belonging to the *Aspergillus* genus. Certain *Aspergillus* species can be characterised by the types of pectinolytic enzymes they are able to produce [10,11,12]. Furthermore, the microbial production of pectinolytic enzymes can be achieved in solid-state fermentation (SSF) or submerged fermentation (SmF), where free and immobilised fungal cells can be used. However, in SSF there are technological problems such as (a) controlling the temperature and pH, and (b) process monitoring due to the nutrient gradients in large scale. Hence, industrial production of enzymes is performed predominantly by SmF [13,14].

Cell immobilisation offers numerous advantages over normal suspended cultures, such as cell stability, higher cell densities, enhanced fermentation productivity, and feasibility of continuous processing. The use of immobilised cells for increasing of enzymatic production has been evaluated increasingly. For example, Nighojkar *et al*. [15] used successfully sodium alginate, glutaraldehyde-treated alginate and polyvinyl alcohol-alginate gel for the immobilisation of whole cells of *A*. *niger*. In this work the authors used dried orange peel powder as inducer for the PG production. The results obtained point out to the possibility of the application of these immobilisation systems in a semi-continuous process for the production of PG.

Shrinivas *et al*. [16] used *Bacillus halodurans* immobilised in a calcium alginate matrix for the production of thermostable alkaline keratinolytic protease in batch and repeated batch cultivation and an increase of 2.5% in the production of protease was obtained compared to free cells after 24 h. Taşkin [17] reported the production of tannase and PG using immobilised cells of *Rhodotorula glutinis* in calcium alginate. These authors obtained a higher production of tannase and PG using the immobilised cells system.

Although different systems for cellular immobilisation have been studied, information about the immobilisation of fungal cells for production of extracellular pectinases remains scarce. The aim of the present study was to establish a rapid method for screening PG from *Aspergillus* strains and employ the most productive strain in a fixed bed reactor using free and immobilised cells with potential industrial applications.

## 2. Results and Discussion

### 2.1. Screening of Aspergillus Strains

Screening is often the first step to select microorganisms with characteristics intended for industrial applications which allows the characterisation and selection of fungal strains with optimal production of enzymes. In addition, the information obtained add-value to these microbial resources preserved in culture collections. Since the fungal enzymes to be used in the food industry must not be contaminated with metabolic toxic compounds, in this study all fungi were previously tested for the presence of mycotoxins (ochratoxin A and fumonisin B_2_), but none of them was considered producers of these metabolites (data not shown).

*Aspergillus* section *Nigri* strains were evaluated for their ability to synthesise PG in microtitre plate wells filled with liquid culture medium (LCM: orange peel 100 g∙L^−1^). The utilisation of orange peel as the unique component of LCM allowed the detection of endo- and exo-PG production. The microtitre plate method has some advantages over other methods of microbial screening. Its main advantage relates to cost effectiveness, since it uses small amounts of culture medium (180 µL); the space occupied by the microtitre plate is limited compared with the space needed when the screening is carried out in Petri dishes or Erlenmeyer flasks and, last but not the least, it is a quantitative method. Currently, there are some developed techniques using a miniaturized approach for production of enzymes such as cellulases and xylanases [18], and a more advanced system which integrates a microbioreactor with a miniaturized continuous separator for enzyme catalyzed reactions [19]. Mrudulab and Anitharaj [20] tested rice bran, wheat bran, sugarcane bagasse, orange peel, lemon peel and banana peel for pectinase production. Orange peel was the substrate that allowed highest pectinase production by *A*. *niger*. Moreover, Maller *et al*. [21] concluded that agro-industrial residues, such as orange and lemon peels, induced production of high levels of PG by *A*. *niveus*. The use of orange peel can be highly economic at the industrial scale.

**Figure 1:**
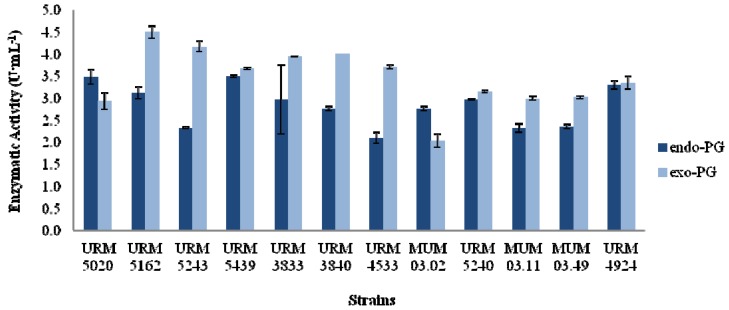
Endo-polygalacturonase and exo-polygalacturonase activities from 12 high producer strains of *Aspergillus* at 72 h.

The increased PG activities were detected between 48 and 96 h. Figure 1 shows the optimal isolates screened for PG production. The maximum endo- and exo-PG activities were obtained by *A*. *niger* strains URM 5439 and 5162, respectively (Figure 1). Looking for both activities simultaneously *A*. *niger* strain URM 5439 shows for endo- and exo-PG the values of 3.5 U·mL^−1^ and 3.67 U·mL^−1^, respectively. It follows that URM 5439 and URM 4924 are the most promising strains to pursue the fermentation studies. These results are in line with those results obtained by Martins *et al*. [22], who evaluated *Thermoascus aurantiacus* in SmF using synthetic media, such as Czapeck, Khanna, SR and Vogel medium supplemented with commercial citrus pectin. The highest activity of PG was obtained in SR (2.0 U·mL^−1^) and Vogel medium (1.9 U·mL^−1^). These higher activities were observed in the logarithmic phase, which occurred after the third or fourth days of the fermentation. Mrudula *et al*. [20] also used agro-industrial by-products as carbon sources. When orange peel was used they obtained two production peaks of production: the first peak occurred after 72 h, and the second at the end of the fermentation at 2.1 U·mL^−1^. Moreover, when passion fruit peel was used, the first peak of production occurred after 72 h (2.0 U·mL^−1^) and the second after 192 h (3.0 U·mL^−1^). In medium containing wheat bran there were two peaks of production after 72 h (2.0 U·mL^−1^) and after 168 h of fermentation (1.8 U·mL^−1^). However, after the fifth day, these 3.2 U·mL^−1^ were obtained when waste from the processing of orange juice was used, which is referred to as yellow water which was the highest activity.

### 2.2. Fixed Bed Reactor

**Figure 2:**
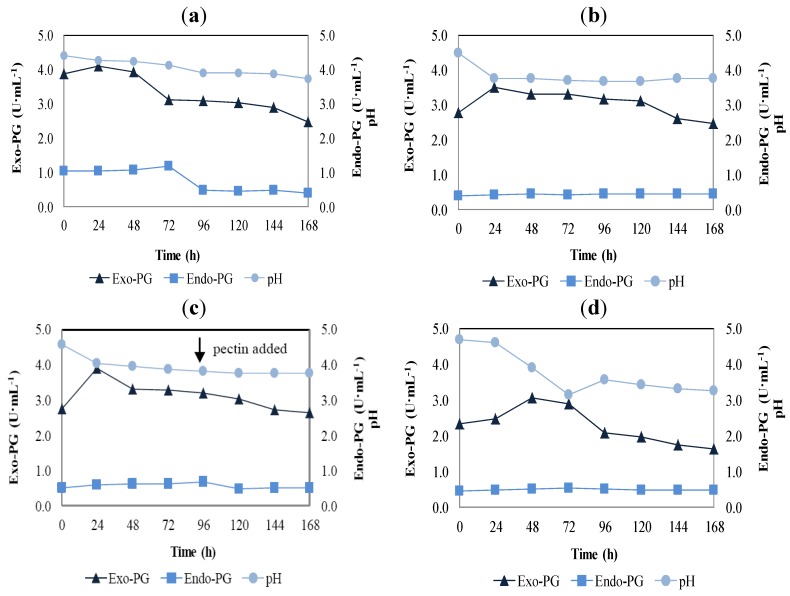
Production of exo-polygalacturonase (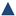) and endo-polygalacturonase (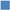) by *Aspergillus niger* strain URM 5162 in fixed bed reactor. (**a**) immobilised cells without aeration; (**b**) immobilised cells with aeration; (**c**) immobilised cells with aeration and adding pectin; and (**d**) free cells with aeration. pH variation during the polygalacturonases production (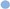).

Figure 2 shows PG activities in the various assays employed. At time zero, the enzymatic activities are due to the substrate being colonised previously with *Aspergillus* spores and incubated for 7 days. The exo- and endo-PG activities reached the maximum values (4.13 U·mL^−1^ and 1.18 U·mL^−1^, respectively) in the assay with immobilised cells without aeration at 24 and 72 h of fermentation, respectively (Figure 2a). However, when compared the assay performed with immobilised cells with aeration (Figure 2b) without aeration (Figure 2a), the PG activities were similar. Non-aeration is advantageous since a reduction in the operation costs is obtained.

The addition of pectin after 96 h of fermentation led to no significant change in the PG activities (Figure 2c). Cordeiro and Martins [23] also observed that increasing the concentration of citrus pectin above 0.5% in the culture medium it did not increase PG activity. The addition of pectin to the fermentation process is an increase in the cost, which often makes the process unviable. The endo-PG activity was kept similar throughout the tests with a small decrease in activity after 96 h. For exo-PG, the highest activity was 3.91 U·mL^−1^ after 24 h of fermentation, followed by a decrease over time (Figure 2c). Higher enzyme production during the first hours of fermentation allows a reduction in the cost of production compared to higher production towards the end.

When compared with the results obtained for the test with free cells (Figure 2d), the cellular immobilisation of *A*. *niger* strain URM 5162 provided a higher activity for exo-PG (Figure 2a–c). However, the endo-PG activities were similar for all tests apart from those up to 72 h in Figure 2(a). Taşkin [17] when worked with PG production by *Rhodotorula glutinis* where the optimal enzyme production was with cells immobilised in calcium alginate beads (28.6 U·mL^−1^). In contrast, the use of free cells led to lower activity (26.9 U·mL^−1^). The culture medium used by this author contained tannic acid, citrus pectin and mineral salts. Gattás *et al*. [24] reported the PG production using an *Aspergillus* sp. immobilised in 3% Ca-alginate. The PG activity obtained was 5.95 U·mL^−1^ in a medium containing 2% glucose and when the medium had no glucose, the PG activity was significantly lower (0.55 U·mL^−1^).

The highest cost of PG production is related to the culture medium supplements, e.g. carbon and nitrogen sources. Gomes *et al*. [8] obtained high production values for PG from supplemented culture media. The maximum PG activity (51.82 U·mL^−1^) was obtained by the use of pectin, L-asparagine, potassium phosphate and iron sulphate at different concentrations (32 g·L^−1^, 2 g·L^−1^, 0.06 g·L^−1^ and 1.0 g·L^−1^, respectively) by SmF with *A*. *niger*. Furthermore, Kumar *et al*. [25] evaluating the production of PG by *A. niger* in SmF, used a mixture of wheat bran, corn bran and kinnow peel in the proportion of 2:1:2, respectively, as carbon source. The maximum enzyme production found was 1.64 U·mL^−1^ using a carbon source concentration of 65 g·L^−1^. Abbasi *et al*. [26] used citrus pectin and wheat flour as carbon source and substrate, respectively, for the production of PG by *A. awamori* in SmF. Initially, the fermentation was carried out in batch until visible fungal growth and then the fermentation regime was changed to continuous feed mode with introduction of fresh medium. These authors obtained a higher production of PG when a concentration of 8 g·L^−1^ of ammonium sulphate as the nitrogen source was used. The production values were 1.5 U·mL^−1^ and 0.014 U·mL^−1^ for exo- and endo-PG, respectively. Furthermore, Galiotou-Panayotou and Kapantai [27] achieved 3.0 U·mL^−1^ of PG in SmF using *A. niger* in medium containing citrus pectin as the carbon source.

Pectinase production has been reported in different types of bioreactors (*i.e.*, stirred tank, airlift and column) [28,29]. According to Kahar *et al*. [30], mycelial growth, morphology and formation of products are related to the type of bioreactor used. In the present work, the pH values of all the samples were monitored. It was observed that the pH of the media was unrelated to PG activities (Figure 2). In this figure a slight pH reduction can be observed in all assays. According to Uenojo and Pastore [31] and Cordeiro and Martins [23] the pH reduction in the fermentation process could be explained by the liberation of glucoronic acid in the medium, because of the action of pectinolytic enzymes. These enzymes are produced by the microorganisms during the first hours of fermentation. According to Ming Chu *et al*. [32], the pH changes in the culture medium can be explained as a result of the substrate consumption. When the ammonium ions are used by the microorganisms the medium is acidified. Furthermore, when the organic nitrogen (amino acids and peptides) are assimilated, the medium is alkalinised. Considering this relationship between the PG and the utilisation of organic compounds, the changes in the pH values could be used to explain some variations in the PG production.

Gomes *et al*. [8] also did not obtain any relationship between the change in pH and the PG production. In addition, Pashova *et al*. [33] working with *A*. *niger* and using 3% Ca-alginate beads for cellular immobilisation, did not observe differences in pH curves between free and immobilised cultures. According to these authors, changes in polymethylgalacturonase (PMG) production could not be explained by pH variation. These authors observed that the fungal immobilisation did not change the model of PMG synthesis. Pashova *et al*. [34] observed that the cellular immobilisation of *A*. *niger* in alginate beads changed the spectrum of pectin-degrading enzymes. The free cell cultures produced four pectinolytic enzymes, namely: PMG, PG, PME and PL, while the entrapped mycelium synthesised PMG and PG only.

### 2.3. Microscopic Observation of Cell Growth in Orange Peels

**Figure 3:**
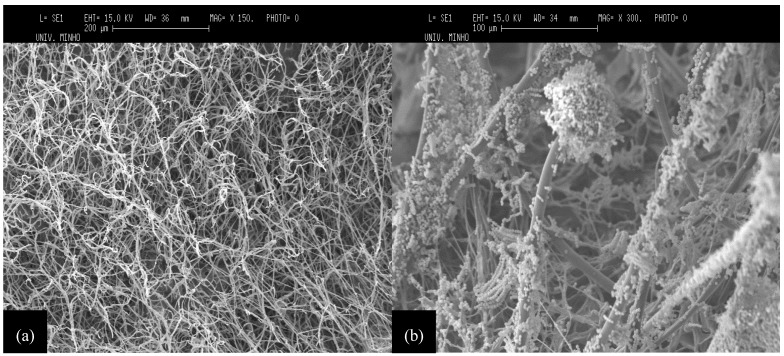
Microphotographs obtained by SEM for orange peel supports colonised by *Aspergillus niger* strain URM 5162: (**a**) support with 4 days of incubation at 25 °C (×150) and (**b**) support with 7 days of incubation at 25 °C (×300).

Figure 3 shows the growth of *A*. *niger* strain URM 5162 on orange peels support. After 4 days of incubation it is possible to observe only the dense mycelium covering the entire surface of the support (Figure 3a). Moreover, a large amount of spores and conidiophores can be observed covering the surface of the support (Figure 3b). The immobilised mycelium was concentrated at the surficial layer of the orange peel support. Pashova *et al*. [33] evaluated the development of *A*. *niger* in calcium-alginate and concluded that alginate gels provide a good environment for the growth of *A*. *niger* cells. According to these authors, the immobilised mycelium became concentrated at the surface layer of the alginate beads and fungal hyphae could not be found in the central part of the gel. In contrast, in the present work the use of orange peel support led to a homogeneous fungal colonisation (Figure 3a).

## 3. Experimental

### 3.1. Microorganisms

Fifty-three isolates of *Aspergillus *section *Nigri* (Table 1) obtained from the Micoteca URM culture collection (URM, Recife, Brazil) [35] and eight fungal isolates of this section obtained from the Micoteca da Universidade do Minho culture collection (MUM, Braga, Portugal) [36] were inoculated on malt extract agar (MEA: malt 20 g L^−1^, glucose 20 g L^−1^, peptone 1 g L^−1^, agar 20 g L^−1^) and incubated for 7 days at 25 °C.

### 3.2. Inoculum Standardisation

Petri dishes with 6 cm of diameter containing 7 mL of sterilised MEA medium were inoculated with each strain and incubated for 7 days at 30 °C. Then, five plugs of 6 mm were cut with a sterile cork borer from the colony periphery to suspend the spores from the plugs in 30 mL of Tween 80-water (0.02%). The spore concentration was adjusted to *c.a.* 3 × 10^5^ spores·mL^−1^.

### 3.3. Miniaturised Screening Method

All strains were subjected to a screening in liquid culture medium (LCM: 100 g of orange peel per litre of distilled water) for evaluation of the PG production. LCM was autoclaved for 15 min at 121 °C. Aliquots (180 µL) of sterile LCM were filled into sterile 96 wells flat-bottom wells polystyrene microtitre plates (Greiner, Frickenhausen, Germany) containing 20 µL of a spore suspension with *c.a.* 3 × 10^5^ spores·mL^−1^ or sterile water (blanks). Once inoculated, microtitre plates were incubated for 5 days at 25 °C and sample were withdrawals every 24 h. The assay was performed in triplicate.

**Table 1:** Strains of *Aspergillus* section *Nigri* evaluated for their ability to produce endo-polygalacturonase and exo-polygalacturonase.

Number	Strains	Number	Strains
URM 13	*Aspergillus niger*	URM 5555	*A*. *niger*
URM 18	*A*. *niger*	URM 5741	*A*. *niger*
URM 19	*A*. *niger*	URM 5837	*A*. *niger*
URM 20	*A*. *niger*	URM 5838	*A*. *niger*
URM 238	*A*. *niger*	URM 5842	*A*. *niger*
URM 949	*A*. *niger*	URM 5910	*A*. *niger*
URM 2228	*A*. *niger*	URM 6054	*A*. *niger*
URM 2813	*A*. *niger*	URM 3452	*A*. *japonicus*
URM 2908	*A*. *niger*	URM 3833	*A*. *japonicus*
URM 3701	*A*. *niger*	URM 3840	*A*. *japonicus*
URM 3753	*A*. *niger*	URM 3916	*A*. *japonicus*
URM 3755	*A*. *niger*	URM 4533	*A*. *japonicus*
URM 3806	*A*. *niger*	URM 4599	*A*. *japonicus*
URM 3811	*A*. *niger*	URM 4663	*A*. *japonicus*
URM 3820	*A*. *niger*	URM 5242	*A*. *japonicus*
URM 3853	*A*. *niger*	URM 5620	*A*. *japonicus*
URM 3856	*A*. *niger*	URM 5633	*A*. *japonicus*
URM 3885	*A*. *niger*	URM 5723	*A. japonicus*
URM 4312	*A*. *niger*	URM 5751	*A. japonicus*
URM 4924	*A*. *niger*	URM 3776	*A. aculeatus*
URM 5020	*A*. *niger*	URM 4953	*A. aculeatus*
URM 5149	*A*. *niger*	URM 5240	*A. aculeatus*
URM 5162	*A*. *niger*	MUM 03.02	*A*. *japonicus*
URM 5207	*A*. *niger*	MUM 03.11	*A*. *aculeatus*
URM 5238	*A*. *niger*	MUM 05.10	*A*. *brasiliensis*
URM 5239	*A*. *niger*	MUM 03.12	*A*. *ellipticus*
URM 5243	*A*. *niger*	MUM 03.49	*A. ibericus*
URM 5253	*A*. *niger*	MUM 06.152	*A. tubingensis*
URM 5437	*A*. *niger*	MUM 06.153	*A. vadensis*
URM 5438	*A*. *niger*	MUM 08.01	*A. uvarum*
URM 5439	*A*. *niger*		

### 3.4. Polygalacturonases Production in Fixed Bed Reactor

#### 3.4.1. Procedures of Immobilisation Biomass on Natural Support

Orange peels were previously sterilised for 2 h in an aqueous solution of 2% sodium hypochlorite (w/v) and then abundantly washed in water. The orange peels were incubated for 36 h at 65 °C for drying and then, exposed to a radiation of ultraviolet light for 2 h in a microbiological cabinet [37]. After dried, 100 g of orange peels were used as supports for immobilisation of the optimal selected strains for PG production. The supports were inoculated with 5 mL of spore suspension containing *c.a.* 3 × 10^5^ spores·mL^−1^. After inoculation, the support was incubated for 7 days at 25 °C for the growth of the fungus. Afterwards, these colonised peels were used as substrate for the fermentation assays.

#### 3.4.2. Scanning Electron Microscopy (SEM)

Observations of the morphological structure of orange peels before and after colonisation by fungal mycelia were assessed by SEM (Leica Cambridge S360, Cambridge, UK). The samples were coated with gold and platinum (80/20%) before microscopic observation.

#### 3.4.3. Submerged Fermentation

**Figure 4:**
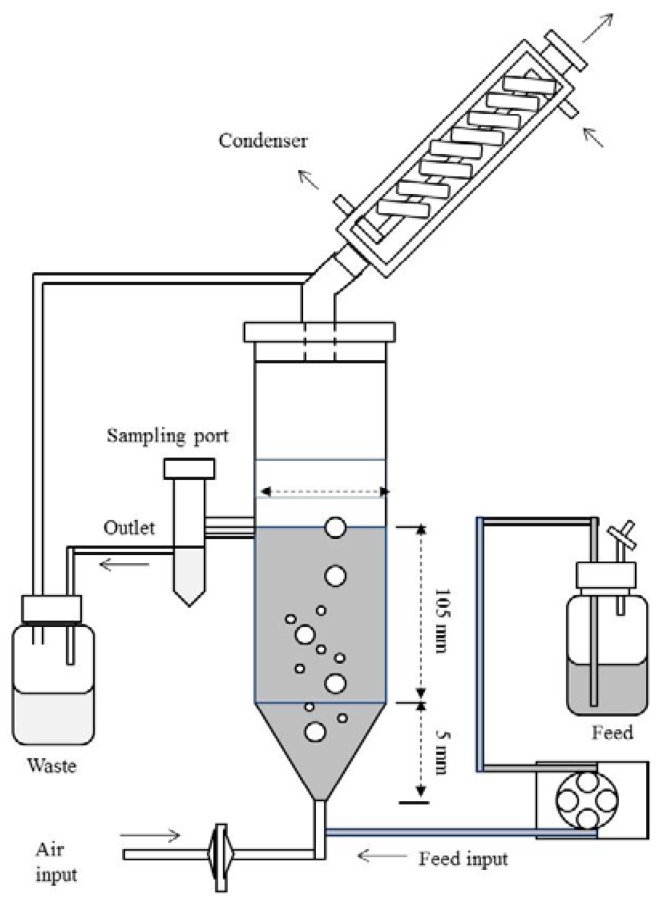
Diagram of the fixed bed reactor used in the present study.

The scale-up of the PG production were carried out in a fixed bed reactor using free and immobilised cells of *A*. *niger* strain URM 5162. A bed reactor of 300 mL in capacity was used with a working liquid volume of 260 mL assisted by a peristaltic pump (Figure 4). In order to maintain fungal metabolism for long periods, continuous LCM addition was performed. The assay was conducted with aeration, which was established by the degree of agitation of the medium avoiding excessive foaming. For fermentation with immobilised cells with aeration pectin (5 g·L^−1^ final concentration in LCM) was added at 96 h in order to maintain fungal metabolism for an extend fermentation period. The PG activities were assessed during 168 h for the assays with free and immobilised cells and samples were withdrawals every 24 h.

### 3.5. Enzymatic Assays

Enzymatic activities of all the samples were expressed in units of activity per liter (U·mL^−1^). Endo-PG activity was measured viscosimetrically by mixing 5.5 mL of 1% (p/v) citric pectin in 0.025 M acetate buffer at pH 5.0 (supplemented with 1 mM EDTA), with 250 μL of the crude of enzymatic extract. The reaction was incubated for 10 min at 50 °C and then cooled in an ice bath. A viscosimetric unit (U) was defined as the enzyme quantity required to decrease the initial viscosity per minute by 50% under the conditions previously described [38]. Exo-PG activity was determined by measuring the release of reducing groups from citric pectin using the 3,5-dinitrosalicylic acid (DNS) assay [39]. The reaction mixture containing 0.5 mL 0.5% citric pectin in 0.025 M acetate buffer, pH 5.0 and 0.5 mL of enzymatic extract was incubated at 50 °C for 10 min. One unit of enzymatic activity (U) was defined as the amount of enzyme which releases one mmol of galacturonic acid per minute.

## 4. Conclusions

In the present study a fast screening methodology for the capacity evaluation of *Aspergillus *section *Nigri* isolates to produce PG was developed. This method was revealed to be appropriate for screening large numbers of fungal strains for PG activities. The highest exo- and endo-PG activities were obtained in the assay with immobilised cells of *A*. *niger* strain URM 5162 without aeration for 24 and 72 h of fermentation, respectively. There was no correlation between PG production and pH of the medium. The process allowed obtained enzyme production in fixed bed reactors.
